# Can IVIG Intervene in AD? Insights from Animal Experiments and Clinical Trials—A Systematic Review and Synthesis Without Meta-Analysis

**DOI:** 10.3390/ijms27052275

**Published:** 2026-02-28

**Authors:** Han Zhao, Zuoming Zhang, Caixian Wang, Fangzhao Lin, Haijun Cao

**Affiliations:** Institute of Blood Transfusion, Chinese Academy of Medical Sciences & Peking Union Medical College, Chengdu 610052, China; s2024019017@student.pumc.edu.cn (H.Z.); s2025019013@student.pumc.edu.cn (Z.Z.); s2024019009@student.pumc.edu.cn (C.W.); lfangzhao@126.com (F.L.)

**Keywords:** Alzheimer’s disease, intravenous immunoglobulin, β-amyloid protein, cognition, encephalatrophy

## Abstract

The clinical safety of intravenous immunoglobulin (IVIG) is well-established, offering potential as a “one-drug, multi-target” intervention for Alzheimer’s disease (AD). However, its efficacy remains inconclusive and appears closely related to specific functional properties. Therefore, we conducted a systematic review based on the analysis of prior animal and clinical trials to provide insights for future IVIG-based therapeutic development. A systematic search was conducted across PubMed, Embase, the Cochrane Library, Web of Science, PsycInfo, ClinicalTrials.gov, SinoMed, and Wanfang databases for the relevant literature published up to 30 October 2025, using terms related to Alzheimer’s, IVIG, and β-amyloid protein. Consequently, IVIG demonstrated clinical safety, though methodologies—including dosages, models, and manufacturers—varied significantly across studies. In most cases, IVIG treatment delayed cognitive degradation in both AD mice and patients. Biologically, Aβ and tau levels increased in plasma while decreasing in the brain or cerebrospinal fluid (CSF), suggesting a peripheral clearance mechanism distinct from that of monoclonal antibody interventions. Additionally, brain atrophy was alleviated, and pathological plaques were reduced. In the context of plasma exchange (PE) combination therapy, the administration of IVIG further contributed to improvements in language, memory, and praxis. IVIG possesses a favorable safety profile and can ameliorate AD symptoms, yet efficacy varies considerably between trials. To advance treatment, future research should investigate the reasons for these variances and establish a standardized system for evaluating preclinical IVIG interventions, thereby facilitating the development of specific IVIG products for AD.

## 1. Introduction

Alzheimer’s disease (AD), a neurodegenerative disorder characterized by insidious onset and progressive decline, features core pathological mechanisms, including aberrant cerebral amyloid-beta (Aβ) deposition [1,2], tau hyperphosphorylation leading to neurofibrillary tangles [3,4], and persistent neuroinflammation [5,6]. As the most prevalent form of dementia in the elderly, AD poses a significant global public health threat. It is reported as the sixth-leading cause of death among individuals aged 65 and older [7]. For decades, AD pharmacotherapy has primarily relied on symptomatic treatments, such as three cholinesterase inhibitors [8,9,10,11,12,13,14,15] and memantine [16,17], which aim to rebalance neurotransmitter dysregulation [18]; however, these agents offer only limited symptomatic relief and cannot alleviate the progression of the disease [19]. More recently, the therapeutic landscape has evolved with the development of second-generation monoclonal antibodies (MABs) targeting Aβ [20,21,22,23,24,25,26,27,28]. While demonstrating some clinical promise in clearing cerebral Aβ plaques and slowing cognitive decline, their clinical utility is constrained by substantial limitations. Most notable is the risk of Amyloid-Related Imaging Abnormalities (ARIA). For Lecanemab specifically, clinical trials reported incidence rates of 12.6% for ARIA-E (edema) and 17.3% for ARIA-H (hemorrhage) [28], particularly in *APOE ε4* carriers [29]. More detailed data from treatment with KISUNLA (donanemab) over a 12-month period showed that, while symptomatic ARIA-E occurred in 3% of patients and symptomatic ARIA-H in less than 1%, the overall incidence rates—including asymptomatic radiographic events—were substantially higher, reaching 29% for ARIA, 16% for ARIA-E, and 25% for ARIA-H [30]. Furthermore, their clinical use is hampered by prohibitive costs, with annual treatment expenses often exceeding $26,500 per patient [31]; with KISUNLA (donanemab) priced at $695.65 per vial, the total cost for a 12-month course of therapy is $32,000 [32]. These collective challenges underscore the urgent need for safer, more effective therapeutic strategies. Consequently, with the escalating global aging population, the number of patients is projected to exceed 139 million by 2050, imposing a substantial socioeconomic challenge [33,34].

Intravenous immunoglobulin (IVIG) is a pooled immunoglobulin concentrate derived from the plasma of thousands of healthy donors [35]. For nearly five decades, IVIG has been extensively utilized to treat various autoimmune diseases, inflammatory conditions, and secondary immunodeficiencies; its long history of clinical application substantiates an established safety profile [36]. Notably, IVIG contains naturally occurring antibodies against Aβ (first reported by Dodel et al. in 2002) [37]; subsequent research has also identified antibodies against tau and Receptor for Advanced Glycation Endproducts (RAGE) [38], as well as soluble low-density lipoprotein receptor-related protein 1 (sLRP1) [39]. A retrospective study found that IVIG therapy was associated with a reduced risk of developing AD and related disorders [40]. These constituents are all thought to beneficially modulate AD pathogenesis. Furthermore, IVIG’s inherent anti-inflammatory properties could effectively counteract AD-related neuroinflammation [41]. Therefore, given its pleiotropic immunomodulatory functions [42], IVIG is regarded as a promising, multi-target therapeutic avenue for AD.

Although preliminary research has yielded encouraging signals [43,44], a scientific consensus on IVIG’s capacity to durably improve cognitive function or modify disease progression in AD patients has yet to emerge. Results from clinical trials were inconsistent [45]. Following the failure of a large Phase III clinical trial by Baxalta (now part of Takeda) [46], global research interest in IVIG for AD intervention appeared to diminish significantly [47].

The reasons for the inconsistent results of clinical trials have rarely been thoroughly investigated. In 2023, Fei et al. found that the efficacy of different IVIGs on AD was significantly different [48], and the efficacy could be positively correlated with its level of AD-related antibodies and anti-inflammatory ability [49]. Therefore, a comprehensive re-evaluation of its therapeutic potential is warranted. This review systematically synthesizes and analyzes the preclinical and clinical literature published through October 2025 to investigate IVIG’s efficacy and safety. We will critically examine its impact on cognitive outcomes, alterations in cerebral Aβ and tau pathology, and structural neuroimaging changes, while also corroborating its established safety profile. The primary objective is to outline the current research landscape and conduct an initial exploration of potential factors contributing to treatment IVIG’s heterogeneity, thereby providing guidance for the development of targeted, next-generation immunoglobulin therapies for AD.

## 2. Research Method

### 2.1. Protocol and Registration

This systematic review was conducted in accordance with the Preferred Reporting Items for Systematic Reviews and Meta-Analyses (PRISMA) 2020 statement. The review protocol for the clinical evidence has been submitted to PROSPERO (Registration number: CRD420261301853), and the comprehensive protocol encompassing both preclinical and clinical evidence has been archived on the Open Science Framework (OSF) (DOI: 10.17605/OSF.IO/4X76T)

### 2.2. Search Strategy

A comprehensive and reproducible systematic search was performed across PubMed, Embase (via Ovid), the Cochrane Library (specifically CENTRAL), Web of Science (Science Citation Index Expanded), PsycInfo, ClinicalTrials.gov, SinoMed, and Wanfang databases from their respective inception to 30 October 2025. To ensure a thorough evidence synthesis, reference lists of the included studies and relevant systematic reviews were manually screened (citation tracing). The search employed a combination of Medical Subject Headings (MeSH) and keywords, including “Alzheimer’s Disease,” “Immunoglobulins, Intravenous,” “IVIG,” “Amyloid beta-Peptides,” and their variants. Detailed search strings for all databases are provided in Supplementary Table S1. No language restrictions were applied to minimize publication bias.

### 2.3. Inclusion and Exclusion Criteria

Studies were selected based on the following criteria:

Participants: For clinical studies, human patients diagnosed with AD or MCI due to AD; for preclinical studies, transgenic mouse models of AD (e.g., 3xTg and APP/PS1).

Intervention: Administration of IVIG (any manufacturer, dosage, or duration).

Comparators: Placebo, vehicle control, or baseline data for self-controlled studies.

Outcomes: Cognitive function (e.g., ADAS-Cog and MMSE), biomarker levels (Aβ, Tau), neuroimaging or pathological changes (brain volume and plaque load), and safety outcomes (adverse events).

Study Design: Randomized controlled trials (RCTs), non-randomized controlled trials, single-arm open-label studies, and controlled animal experiments.

Exclusion criteria: reviews, editorials, case reports, conference abstracts without complete data, and studies utilizing non-standard routes of administration (e.g., subcutaneous) without an IVIG comparison group.

### 2.4. Data Extraction and Risk of Bias Assessment

#### 2.4.1. Data Selection and Extraction

Two reviewers (H.Z. and Z.Z.) independently screened titles/abstracts and full texts. Disagreements were resolved by discussion or consultation with a third reviewer (H.C.). Extracted data included study characteristics, participant/animal model details, intervention regimens, and outcomes.

#### 2.4.2. Quality Assessment

The Risk of Bias Was Assessed Independently by Two Reviewers.

For animal studies, the SYRCLE’s RoB tool (https://link.springer.com/article/10.1186/1471-2288-14-43 was applied, accessed on 7 January 2025) was utilized to evaluate selection, performance, detection, attrition, and reporting biases.

For clinical RCTs, the Cochrane RoB 2 tool (https://sites.google.com/site/riskofbiastool/welcome/rob-2-0-tool/current-version-of-rob-2, accessed on 7 January 2025) was applied.

For non-randomized/single-arm clinical studies, the ROBINS-I-V2 tool (https://sites.google.com/site/riskofbiastool/welcome/robins-i-v2. accessed on 7 January 2025) was employed. The certainty of evidence for clinical outcomes was further evaluated using the Grading of Recommendations Assessment, Development and Evaluation (GRADE) (https://www.gradepro.org/, accessed on 7 January 2025) approach.

### 2.5. Data Synthesis and Analysis

Given the substantial heterogeneity in study designs, animal models, and dosing regimens, a quantitative meta-analysis was deemed inappropriate. Instead, a narrative synthesis was performed following the Synthesis Without Meta-analysis (SWiM) reporting guidelines. Data were categorized by population (preclinical vs. clinical) and study design. Treatment effects were synthesized using mean differences or direction-of-effect (improvement, no change, or decline), with *p*-values reported where applicable. Heterogeneity was qualitatively explored by analyzing variations in IVIG products, dosages, and AD stages.

### 2.6. Assessment of Certainty of Evidence

The certainty of evidence for clinical outcomes was evaluated using the Grading of Recommendations Assessment, Development and Evaluation (GRADE) approach. Two reviewers independently assessed five domains: risk of bias, inconsistency, indirectness, imprecision, and publication bias. The certainty was categorized into four levels: high, moderate, low, and very low.

## 3. Results

### 3.1. General Information Included in the Study

**Figure 1:**
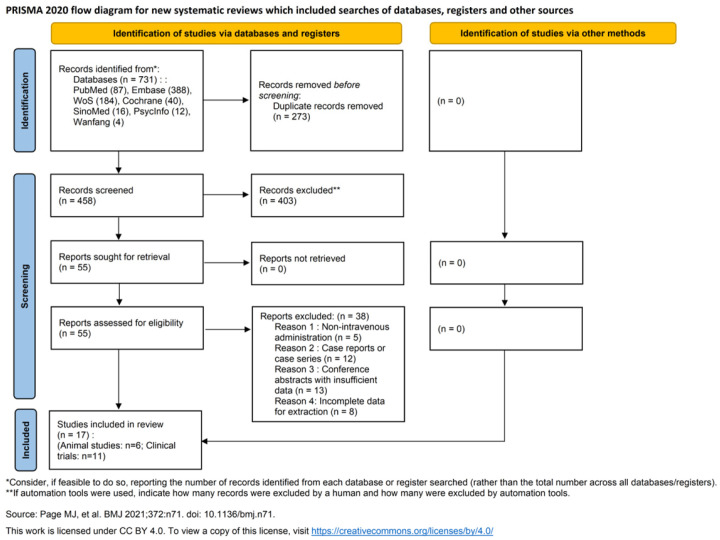
PRISMA 2020 flow diagram of the study selection process.

A total of 731 articles were retrieved, with 17 being included following rigorous screening, including 6 animal experiments and 11 clinical studies (14 articles); among 11 clinical studies, 8 analyzed the efficacy of IVIG (9 articles), 8 examined safety (9 articles), and 1 investigated combined IVIG and PE therapy (3 articles) (Figure 1).

The basic information of six animal experiments is shown in Table 1. Across six animal experiments, five articles were published in 2012–2014 [50,51,52,53,54]. All studies utilized Aβ transgenic mouse models, but the specific mouse strains varied, including 3xTg-AD mice [48,51,52], AβPP mice [50] and APP/PS1 mice [53,54]. The IVIG manufacturers were reported as Bayer (Pittsburgh, PA, USA) [50], Gamunex™ (Grifols Canada Ltd., Mississauga, ON, Canada) [51], and Gammagard 10% (Baxter Healthcare, Deerfield, FL, USA) [52], while, in other studies [48,53,54], the manufacturer was not specified. The injection site included abdomen [48,51,54], retro-orbital sinus [52], bilateral intracranial [53] and vein [50]. The injection doses, frequency and cycle were also inconsistent.

The basic information of eight clinical studies that analyzed the efficacy is shown in Table 2. Across nine clinical studies that analyzed the efficacy, the publication year was from 2002 to 2021, which was wider than animal research. The number of patients included ranged from 5 to 390. Although the IVIG manufacturer injection dose and frequency were also inconsistent, 6/9 experiments used Octagam [37,44,55,56] or Gammagard [57,58]; the injection dose for the 4/9 experiment was 0.4 g/kg, administered every two weeks [55,57,59,60,61]. The treatment period ranged from 10 to 72 weeks, with 10 weeks in the majority.

### 3.2. Risk of Bias Assessment

**Figure 2:**
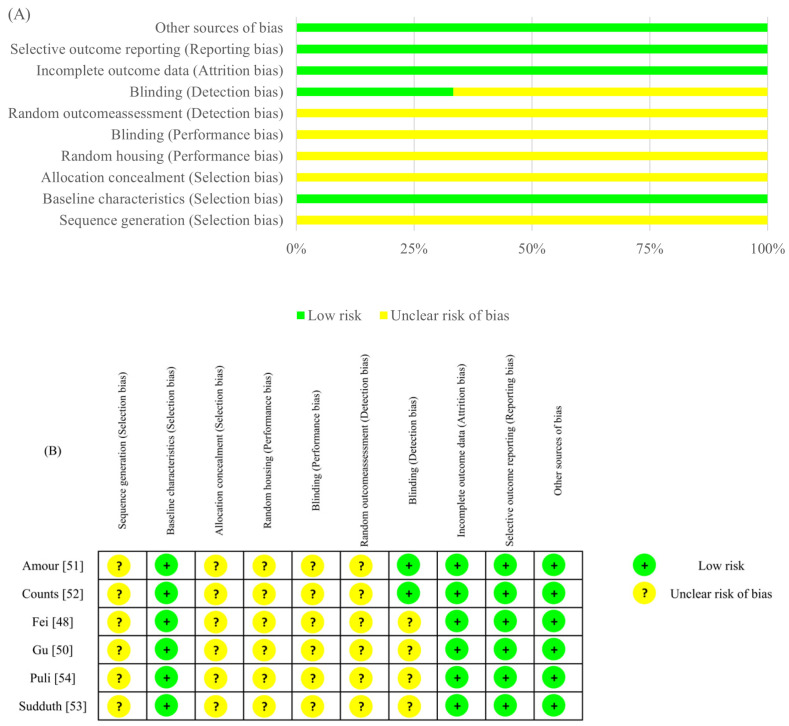
Risk of bias assessment for animal studies using the SYRCLE tool [62]. (**A**) Summary bar chart representing the percentage of studies with low, unclear, or high risk of bias for each domain. (**B**) Traffic light plot showing the risk of bias for each individual included study [48].

**Figure 3:**
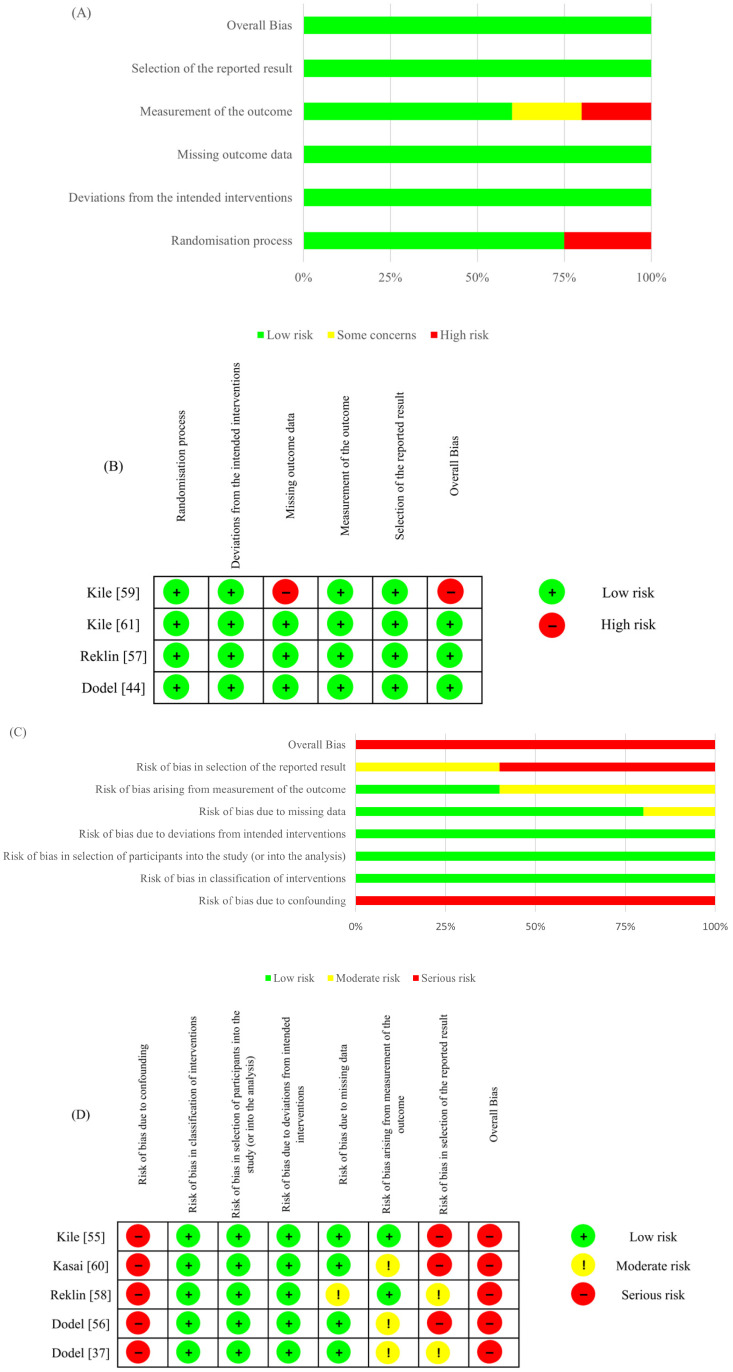
Risk of bias assessment for clinical studies. (**A**) Summary risk of bias for RCTs across each domain. (**B**) Individual study risk of bias for randomized controlled trials (RCTs) using the Cochrane RoB 2 tool. (**C**) Summary risk of bias for non-randomized evidence across each domain. (**D**) Individual study risk of bias for non-randomized clinical studies using the ROBINS-I-V2 tool [63,64].

The methodological quality of the included studies was assessed to ensure the reliability of the evidence. The results are summarized in Figure 2 (preclinical) and Figure 3 (clinical). For preclinical studies (SYRCLE), a high prevalence of “unclear risk” was observed in domains related to randomization procedures and blinding, reflecting a general need for improved reporting standards in animal research (Figure 2). Regarding clinical RCTs (RoB 2), most studies exhibited a low overall risk, though Kile [59] was identified as high risk due to missing outcome data. Conversely, non-randomized studies (ROBINS-I) consistently showed a serious risk of bias, primarily stemming from potential confounding and selective reporting (Figure 3).

### 3.3. Animal Experiments

Animal behavioral tests are important tools in neurobiological studies to assess learning and memory abilities, anxiety, depression, fear and locomotion. However, in the above studies, only two studies conducted behavioral tests (Table 3). In Fei et al. [48] study, 3-month-old 3xTg-AD mice were randomly divided into three groups and administered intraperitoneally with different IVIG (A/B/C) for 3 months, and then behavioral tests were conducted at 9 months old. Compared to the group receiving physiological saline infusion, all groups improved the motor and autonomous decline in the open-field experiment test, and only IVIG-C ameliorated cognitive and motor decline in NOR test and Barnes maze test. However, the behavioral test outcomes of Amour et al. [51] study were not consistent with Fei et al. In Amour et al. [51] experiment, behavioral tests were conducted on 12- or 16-month-old 3xTg-AD mice after the administration of IVIG for 1 or 3 months. The NOR index assessed in 16-month-old animals for both treatment durations was significantly ameliorated. The anxiety-like behavior in the dark–light box emergence test in 12-month-old mice was mitigated. And the results of the open-field experiment and Barnes maze experiment showed no significant difference between AD mice treated with IVIG and untreated non-transgenic mice. The two studies suggest that IVIG had an improvement effect on the cognitive ability of AD mice. However, the efficacy differed across experimental groups, which could be related to variations in IVIG manufacturers, the age of the mice, and the duration of treatment.

There were five studies that detected Aβ_40_ and Aβ_42_ levels in plasma or brain in AD model mice after the administration of IVIG (Table 3). The total of Aβ in plasma was detected only in Gu et al. [50] study, and the result showed that, compared to the control group, the total of Aβ in the experimental group decreased in plasma and increased in the brain. In Sudduth et al. [53] study, Aβ_40_ and Aβ_42_ levels in the right frontal cortex were decreased. And, in Fei et al. [48] study, only soluble Aβ_40_ in the parietotemporal cortex of the IVIG-C group was decreased, and only the behavior of the IVIG-C was improved in the open-field experiment test, NOR test and Barnes maze test. However, in Puli et al. [54] study, soluble Aβ_40_ and Aβ_42_ in the hippocampus were increased in APP mice after 32 weeks of IVIG injection. Aβ_40_/Aβ_42_ could also change after IVIG injection; in Amour et al. [51] study, soluble Aβ_40_/Aβ_42_ in the parietotemporal cortex were decreased in 16-month-old 3xTg-AD mice, and only the groups with reduced Aβ_42_/Aβ_40_ showed an improved NOR index.

There were five studies that detected pathological plaques in AD mice after the administration of IVIG (Table 3). On the whole, the changes in Aβ deposition in the mouse brain were consistent with the changes in Aβ levels in the brain. Fei et al. [48] study showed that the deposition of Aβ was alleviated and the amount of p-tau was reduced in the hippocampus of 3xTg-AD mice by IVIG-C. The deposition of Aβ was alleviated in Sudduth’s [53] study as well. Count et al. [52] study showed that the amount of tau was reduced in the CA1 Pyramidal Neurons. However, there were no significant changes in the deposition of Aβ and the amount of p-tau in parietotemporal cortices in Amour’s study, and the deposition of Aβ in the hippocampus in Puli et al. [54] study.

### 3.4. Clinical Trials

There were six studies that evaluated the cognitive scale of patients after treatment, including Alzheimer’s Disease Assessment Scale-Cognitive Subscale (ADAS-Cog), Mini-Mental State Examination (MMSE), Clinical Dementia Rating Scale Sum of Boxes (CDR-SB), and others (Table 4). In all the studies of cognitive scale evaluation, only ADAS-Cog and MMSE could change after IVIG treatment, and other indicators had no significant change. The studies by Kasai et al. [60], Kile et al. [61], and Dodel et al. [56] showed that ADAS-Cog was decreased and MMSE was increased. And, in Relkin et al. [58] study, MMSE was increased after treatment. However, in other studies, there was no significant change in the cognitive scale scores of patients after treatment.

There were six studies that detected Aβ_40_ and Aβ_42_ in plasma or CSF in AD patients after the administration of IVIG (Table 4). Patients with Aβ increased in plasma or decreased in CSF had an improvement on cognitive performance [56,58,60]. This pattern is distinct from that observed in patients undergoing monoclonal antibody therapy. Specifically, after injecting Lecanemab in AD patients, Aβ_40_ and Aβ_42_ levels were observed to decrease in plasma and increase in CSF [28]. Within the IVIG studies, the changes in plasma Aβ levels were more pronounced than those in CSF.

In four studies, Aβ increased in plasma and decreased in CSF. In particular, Aβ in peripheral plasma seemed to be a more direct and precise biomarker than Aβ in plasma [60]. However, Aβ_42_ in plasma was decreased in Relkin et al. [57] and Dodel et al. [44] studies. In addition, the changes in Aβ levels after IVIG injection in recent years are not as obvious as those of more than 10 years ago.

Since histochemical analysis of brain tissue is not feasible in clinical trials, imaging methods such as Positron Emission Tomography-Computed Tomography (PET-CT) or Magnetic Resonance Imaging (MRI) are used to observe brain atrophy or retinal amyloid deposition. Annualized percent change in ventricular volume (APCV) was the most commonly used indicator among them [57,59,61]. There were six studies with imaging-related detection, and three of them had changes (Table 4). Kile et al. [59] detected APCV between baseline and 5 years, and the results showed that the effects of IVIG were most pronounced in L-MCI. And, in Kile et al. [61] previous study, APCV was significantly lower compared with the control group. In addition, Kile et al. [55] study showed that three subjects had a reduction in amyloid standard uptake value ratio (SUVR), and all five subjects had a reduction in amyloid retinal deposits in the eyes. However, in studies where cognitive function had been improved, there could not be significant changes in imaging examination [60].

### 3.5. Plasma Exchange Combination IVIG

In addition to using IVIG alone for intervention, there are also combinations of IVIG with other methods. Boada et al. [65,66,67] conducted a phase 2b/3 trial, which examined the effects of plasma exchange (PE) in patients with mild-to-moderate AD (Table 5). There were three PE-treatment groups: low-albumin groups, low-albumin and IVIG groups, and high-albumin and IVIG groups. PE-treated patients performed significantly better than placebo in cognitive performance, the AD Cooperative Study-Activities of Daily Living (ADCS-ADL) showed 52% less decline in PE-treated compared to placebo patients (*p* = 0.03) from baseline to month 14, while the AD Assessment Scale-Cognitive Subscale (ADAS-Cog) showed 66% less decline (*p* = 0.06). All treatment groups showed significantly less decline compared to placebo in the CDR-sb, while ADCS-CGIC scores were stable in all treatment groups at month 14. In all treatment groups, levels of Aβ_42_ and tau protein in CSF remained stable. However, in the placebo groups, levels of Aβ_42_ and tau protein in CSF were decreased in mild AD and increased in moderate AD. The changes in Aβ_42_ and tau protein levels in CSF were particularly evident in moderate AD patients but inconclusive or even counterintuitive in mild AD patients. Although cognitive function improved in each PE-treated group, no promoting effect of IVIG on cognitive function improvement was found from the cognitive scores. However, from the outcomes of neuropsychological and neuropsychiatric in AD patients, IVIG promotes improvements in language, memory, and praxis.

### 3.6. Safety of IVIG Treatment for AD

There is no animal experimental study on the safety of IVIG in the treatment of AD. However, at present, no adverse reactions have been reported in all animal experiments. As to the clinical studies, there are sufficient experiments (Table 6), including congestive cardiac failure, myocardial infarction, and vasogenic cerebral edema. Although some patients in the study had adverse reactions after IVIG treatment, no serious adverse reactions were related to IVIG.

In addition, some systematic reviews had evaluated the safety of IVIG treatment in AD patients, and all studies indicate that IVIG is safe [70,71,72,73]. Three plasma administrations and five IVIG randomized controlled trials were included in Fei et al. systematic review, and they performed a safety meta-analysis [70]. In Liu et al. [71] meta-analysis (755 patients in five RCTs), the number of patients with adverse events did not differ between IVIG and placebo groups (RD −0.00, 95% CI −0.05 to 0.05, *p* = 0.89). In general, IVIG is safe and well-tolerated.

### 3.7. Summary of Findings and Certainty of Evidence

The GRADE “Summary of Findings” for primary clinical outcomes is presented in Table 7. In brief, the evidence supporting IVIG’s safety and lack of ARIA risk was of high certainty. However, the evidence for cognitive improvement (ADAS-Cog/MMSE) was of low certainty, primarily downgraded due to the inclusion of small-scale open-label studies and the inconsistent results between early-phase trials and larger Phase III investigations.

## 4. Discussion

The incidence of AD exhibits an age-dependent increase, with the peak onset occurring in individuals aged 65 years and above [7]. The risk of developing the condition is highest among those aged 85 years and above [74]. Consequently, the safety profile of medications is of paramount importance. The recent FDA approvals of MABs, such as Lecanemab, had established a new benchmark for AD therapy [75]. These drugs operate via a highly specific and potent mechanism: directly targeting and clearing cerebral Aβ aggregates [28,76]. This potent, “brute-force” plaque removal, however, is mechanistically linked to a significant safety liability, ARIA, which presents a considerable risk, particularly for APOE4 carriers [29]. In contrast, preclinical studies, individual clinical trials, and meta-analyses had consistently demonstrated that IVIG intervention in AD exhibits a favorable tolerability and safety profile. From a safety standpoint, IVIG presents a compelling therapeutic option for AD.

A key strength of this review is the transparent assessment of evidence using the GRADE approach. Our findings highlight a stark contrast between the high-certainty evidence for IVIG’s safety and the low to moderate certainty for its clinical efficacy. The low certainty in cognitive outcomes is largely attributed to the “neutral findings” of large-scale RCTs compared to smaller exploratory studies. These limitations underscore the need for more standardized IVIG products with specific antibody titers against Aβ or tau, as suggested by the heterogeneity discussed earlier.

Despite its established safety [59,77], the efficacy of IVIG in AD treatment showed significant heterogeneity by both preclinical and clinical research. This observed inconsistency led to several systematic reviews and meta-analyses which, after pooling data from disparate trials, largely concluded that IVIG lacks overall efficacy for AD [45]. Although research into the use of IVIG for AD has spanned 21 years, yet to date no IVIG product has been approved for the treatment of AD [56,78]. Our findings prompt a critical re-evaluation of this conclusion: does this pooled “null finding” reflect a true class-wide failure, or does it mask the potential efficacy of specific, high-potency formulations?

The conclusions on efficacy from these meta-analyses are likely confounded by substantial methodological heterogeneity, including the IVIG manufacturer [49], injection dose, frequency and duration of treatment. This review argues that a key flaw in previous aggregate analyses is the implicit assumption that all IVIG products are therapeutically interchangeable. This premise is directly challenged by emerging preclinical evidence. Most notably, a study by Fei et al. reported that different commercial IVIG products exhibited differential neuroprotective effects in 3xTg-AD mice; among the three manufacturers tested, only one conferred significant neuroprotective benefits, which was correlated with the downregulation of proteins associated with antigen processing [48,67]. This finding clearly demonstrates a link between composition and therapeutic effect, strongly suggesting that the therapeutic potential of IVIG is manufacturer-specific and formulation-dependent. Given the complex composition and diverse mechanisms of action of IVIG, products from different manufacturers vary in their specific constituents, leading to variable treatment outcomes. Therefore, the heterogeneity observed in clinical trials is likely not random noise but a direct consequence of product-specific biochemical differences, such as varying anti-Aβ antibody titers and other immunomodulatory components [79].

Aβ antibody could be an important component of IVIG in the treatment of AD, so it is necessary to develop a specific IVIG for the treatment of AD. Although specific products had been proposed for a long time [80], they had not been implemented. The specific constituents and efficacy vary between different IVIG manufacturers, leading to differences in therapeutic outcomes [37,41]. Due to the complex composition and functional mechanism of IVIG and the differences in composition and function between different manufacturers [81], it is necessary to carry out a fundamental study of IVIG’s intervention mechanisms on AD. IVIG is derived from healthy human plasma, but the plasma of different populations had an important impact on the composition [82,83]. Although the preparation process has a certain impact [84], the plasma donor factor cannot be ignored. It has been found that there are significant differences in Aβ levels in IVIG from different manufacturers [79,85]. Compared with European Octagam (Octapharma AG, Vienna, Austria), the content of Aβ in Chinese products is generally higher. Aβ_40_ monomer, Aβ_40_ soluble oligomers, Aβ_42_ monomer and Aβ_42_ soluble oligomers’ concentrations in Chinese IVIG preparations were 16.53, 8.47, 24.36 and 33.25 μg/mL, while in Octagam IVIG were 1.66, 2.07, 4.61 and 4.64 μg/mL [79]. In the previous study, it was found that there are great differences in Aβ antibody levels among different individual plasma donors and regional donor groups in China (unpublished data), which provides conditions for preparation of specific IVIG products. In addition, the average ex-factory price of IVIG in China is about 220–280 dollars/10 g, while the price of IVIG in the United States is about 800–1000 dollars/10 g, and specific products also have advantages over monoclonal antibodies in terms of price. In summary, it is feasible for China to develop specific IVIG products to prevent AD.

Both animal studies and clinical trials demonstrate variations in the efficacy of different IVIG interventions for AD. Consequently, the effectiveness of IVIG in treating AD cannot be assessed holistically, nor should it be categorically dismissed based on the failure of a single phase III clinical trial [49,57]. This also underscores the critical importance of determining the optimal drug dosage and timing to maximize therapeutic efficacy [86,87]. In summary, it reframes the narrative from one of universal inefficacy to one of profound heterogeneity, demanding a more nuanced, product-specific approach to future clinical evaluation.

**Figure 4:**
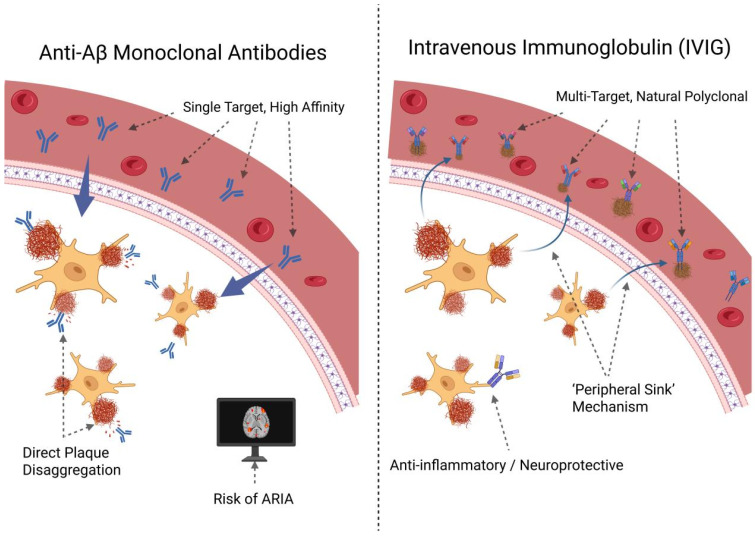
Comparison of mechanisms of action between monoclonal antibodies and IVIG in the treatment of AD. Image created on the biorender: https://BioRender.com/ jhy1bz8 (accessed on 25 November 2025).

Existing data from animal and clinical trials on IVIG for AD showed similar, largely consistent trends toward improvement. In most study findings, after IVIG treatment, the degradation process in the cognitive function of AD mice or AD patients was delayed. At the same time, the levels of Aβ and tau in plasma increased, the levels of Aβ and tau in the brain or CSF decreased, and the pathological plaques in brain tissue reduced or brain atrophy alleviated. In the IVIG studies discussed herein, while cognitive function improved, the levels of Aβ and tau were decreased in CSF and increased in plasma. Therefore, it is speculated that effective intervention was associated with decreased CSF Aβ and increased plasma Aβ. The effects of IVIG intervention in AD differ from those of monoclonal antibodies. In stark contrast, IVIG offers a fundamentally different therapeutic paradigm. As a natural polyclonal preparation, it functions as a multi-target agent. Its therapeutic potential is not limited to a single pathway but encompasses naturally occurring antibodies against Aβ, tau, and RAGE, alongside broad anti-inflammatory and immunomodulatory properties [37,38,39,41]. This multi-pronged, potentially homeostatic mechanism likely explains IVIG’s most significant advantage over MABs: its superior and long-established safety profile [36]. This mechanistic divergence is vividly reflected in their respective biomarker signatures; whereas potent MABs induce a rapid and substantial decrease in brain amyloid load detectable by PET imaging, IVIG administration consistently results in a more subtle pattern—as shown in several trials reviewed here—of increased plasma Aβ and decreased CSF Aβ. This signature strongly supports the “peripheral sink” hypothesis, suggesting IVIG facilitates a gentle efflux of Aβ from the brain into the circulation [88,89], rather than aggressive in situ plaque disruption. This “gentle” clearance model provides a compelling biological rationale for why ARIA is not a characteristic side effect of IVIG therapy. In essence, whereas MABs aim to remove a pathological hallmark, IVIG could function by restoring physiological homeostasis, positioning it as a potentially safer therapeutic alternative. A diagram comparing the mechanisms of action of MABs and IVIG in the treatment of AD is also provided (Figure 4).

**Figure 5:**
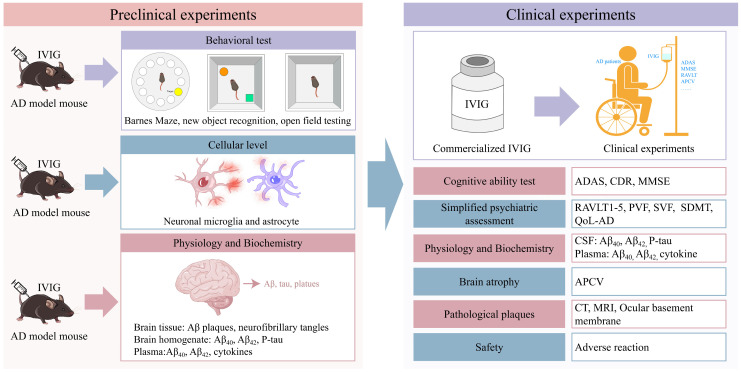
Preclinical and clinical research process for IVIG treatment of AD. Image created on PowerPoint and Adobe Illustrator 2024.

Since many research’s manufacturer and other information was not provided or hidden, this study failed to summarize and compare the efficacy of various manufacturers. Although there had been more than a dozen evaluations on the efficacy of IVIG on AD, few studies had directly evaluated cognitive function both in animal and clinical studies. Future studies should comprehensively evaluate cognitive function, physiology, biochemistry, pathology and other aspects. In addition, preclinical research is an important basis for clinical studies. At present, there are few animal experiments on the efficacy of IVIG on AD, especially after 2014. Nowadays, there are advanced foundations and technologies to carry out AD-related animal research, transgenic animal models [90], behavioral tests [91,92,93], physiological and biochemical tests, molecular biology testing [94], and tissue immune detection technology. Given that past preclinical and clinical studies had not been well-aligned and study designs had often been incomplete, it is necessary to establish a comprehensive research framework for IVIG in the treatment of AD (Figure 5).

Furthermore, the combination of IVIG with other interventions for AD warrants further exploration. Boada et al. phase 2b/3 clinical trial is the only clinical trial of plasma exchange combined with IVIG injection in the treatment of AD. Although IVIG promotes improvements in language, memory, and praxis, the cognitive function, physiological and biochemical indexes, mental state and quality of life in the treatment group were improved compared with those of the placebo group, no differences were found among the three treatment groups, and the effect of IVIG on the efficacy was not clear [65,66,67,95]. Dubey et al. [96] discovered that enhancing blood–brain barrier permeability via focused ultrasound to deliver effective doses of IVIG to the hippocampus promotes neurogenesis in an AD mouse model. In the course of investigating IVIG’s therapeutic effects on AD, exploring combinations with other technical modalities could lead to more suitable treatment regimens.

## 5. Conclusions

Based on findings from animal studies and clinical trials, IVIG exhibits a favorable safety profile. In conclusion, IVIG could alleviate symptoms of AD, including improvements in cognitive function and disease progression delay, though the efficacy varies across different studies.

Future research should pay attention to the heterogeneity of IVIG intervention on AD and focus on preclinical studies, conducting comprehensive assessments across multiple domains, including cognitive function, physiology, biochemistry, and pathology. Investigating the causes of therapeutic efficacy variations inherent to IVIG therapy itself, alongside developing targeted preventative and specific IVIG, represents a key direction for future advancement. Additionally, combining IVIG with other approaches for treating AD could also prove a viable strategy.

## Figures and Tables

**Table 1:** The basic information of 6 animal experiments.

Publication Year	First Author	Experimental Animals	Brand	Injection Site	Age of Initial Injection	Dose	Control Group	Cycle	Age of Testing Time
2023	Fei [48]	3xTg-AD mice (*n* = 14/group)	Three kinds of IVIG	i.p.	12 weeks	1 g/kg, twice a week	NS (AD, *n* = 10); Untreated (WT, *n* = 10)	12 weeks	36 weeks
2014	Gu [50]	AβPP mice (*n* = 6)	Bayer	i.v.	12 weeks	0.02 g/kg/week	IVIG without anti-Aβ (AD, *n* = 6); NS (AD, *n* = 6)	2 weeks	16 weeks
2014	Counts [52]	Female 3xTg-AD mice (*n* = 15/group)	Gammagard	r.o.s.	12 weeks	0.4 g/kg/2 weeks	10% Sigma (AD, *n* = 15); Untreated (AD, *n* = 8)	12 weeks	36 weeks
								24 weeks	48 weeks
2014	Amour [51]	3xTg-AD mice (mixed, *n* = 38)	Gamunex	i.p.	36 weeks	1.5 g/kg, twice a week	0.2 M glycine pH 4.25 (AD, *n* = 31; WT, *n* = 24) IVIG (WT, *n* = 8)	12 weeks	48 weeks
					52 and 60 weeks			12 or 4 weeks	64 weeks
2013	Sudduth [53]	APP/PS1 mice (*n* = 30)	Not mentioned	i.c.v.	28 weeks	0.002 mg per mouse	NS (AD, *n* = 30); anti-Aβ antibody (AD, *n* = 30); mouse IgG (*n* = 30)	Once	29 weeks + 1, 3, 7, 14 and 21 days
2012	Puli [54]	APP/PS1 mice (*n* = 16/group)	Not mentioned	i.p.	16 weeks	1 g/kg/week	NS (AD, *n* = 15)	12 weeks	39 weeks
								32 weeks	49 weeks

Note: WT = Wild Type; AD = Alzheimer’s Disease model; NS = Normal Saline; i.p. = Intraperitoneal Injection; i.v. = Intravenous Injection; r.o.s. = Retro-orbital Sinus Injection; i.c.v. = Intracerebroventricular Injection (bilateral intracranial).

**Table 2:** The basic information of 9 clinical studies that analyzed the efficacy.

Publication Year	First Author	Experimental Subjects	Brand	Dose	Control Group	Cycle	Test Time
2021	Kile [59,61]	50 patients with amnestic MIC	Not mentioned	0.4 g/kg/2 weeks	NS (*n* = 25)	10 weeks	1, 2, 3, 4, and 5 years after completing treatment
2015							
2020	Kile [55]	5 MCI patients	Octagam	0.4 g/kg/2 weeks	Not applicable	10 weeks	3 months after completing treatment
2017	Kasai [60]	5 patients with AD	Venoglobulin	0.4 g/kg, 3 time per 4 weeks	Not applicable	8 weeks	Baseline and before each cycle of IVIG
2017	Relkin [57]	390 patients with mild to moderate AD	Gammagard	0.2 or 0.4 g/kg every 2 weeks	Low-dose albumin (*n* = 130)	72 weeks	Baseline and every 3 months through month 18
2013	Dodel [44]	89 patients with mild to moderate AD	Octagam	0.2 g/0.5 g/0.8 g/kg, 4 times a week (*n* = 22); 0.1 g/0.25 g/0.4 g/kg, twice a week (*n* = 21)	NS (*n* = 8)	24 weeks	Baseline and at 6 months
2009	Relkin [58]	8 patients with mild AD	Gammagard	0.4 g/kg/2 weeks, 0.4 g/kg/weeks, 1 g/kg/2 weeks, 2 g/kg/4 weeks	Not applicable	24 weeks	Baseline and at 3-month intervals thereafter
2004	Dodel [56]	5 patients with AD	Octagam	0.4 gm/kg, 3 times per 4 weeks	Not applicable	24 weeks	Baseline and 6 months following IVIG
2002	Dodel [37]	7 patients with AD	Octagam	0.4 gm/kg, 3 consecutive days	Not applicable	3 days	The indicated times after the infusions

Note: AD = Alzheimer’s Disease; MCI = Mild Cognitive Impairment; NS = Normal Saline.

**Table 3:** The efficacy of IVIG in AD in animal experiments.

First Author	Age of Testing Time	Open-Field Experiment Test	Novel Object Recognition Test	Barnes Maze Test	Light–Dark Box Emergence Test	Aβ_40_ in Plasma	Aβ_42_ in Plasma	Aβ_40_ in Brain	Aβ_42_ in Brain	Aβ_42_/Aβ_40_ in Brain	Aβ Deposition in Brain	Tau Deposition in Brain
Fei [48]	36 weeks-A	↑	O	O	N	N	N	O parietotemporal cortex	O parietotemporal cortex	O parietotemporal cortex	O hippocampus	O hippocampus
	36 weeks-B	↑	O	O	N	N	N	O parietotemporal cortex	O parietotemporal cortex	O parietotemporal cortex	O hippocampus	O hippocampus
	36 weeks-C	↑	↑	↑	N	N	N	↓ parietotemporal cortex	O parietotemporal cortex	O parietotemporal cortex	↓ hippocampus	↓ hippocampus
Gu [50]	16 weeks	N	N	N	N	↑ total		↓ total		N	N	N
Counts [52]	36 or 48 weeks	N	N	N	N	N	N	N	N	N	N	↓ CA1 Pyramidal Neurons
Amour [51]	48 weeks	N	O	N	↑	N	N	O parietotemporal cortex	O parietotemporal cortex	O parietotemporal cortex	O parietotemporal cortex	O parietotemporal cortex
	64 weeks	O	↑	O	N	N	N	O parietotemporal cortex	O parietotemporal cortex	↓ cytosolic fraction	O parietotemporal cortex	O parietotemporal cortex
Sudduth [53]	29 weeks + 1, 3, 7, 14 and 21 days	N	N	N	N	N	N	↓ hippocampus	↓ hippocampus	N	↓ frontal cortex and hippocampus	N
Puli [54]	39 weeks	N	N	N	N	O	O	↑ hippocampus	↑ hippocampus	N	O hippocampus	N
	49 weeks	N	N	N	N	O	N	O hippocampus	O hippocampus	N	O hippocampus	N

Note: ↑/↓ = statistically significant increase/decrease (*p* < 0.05). O = no significant difference. N = not reported. Open-Field Experiment Test: the distance of movement. Novel Object Recognition Test: time in exploring the novel object. Barnes Maze Test: time of staying in the target zone. Light–Dark Box Emergence Test: time in light box.

**Table 4:** The efficacy of IVIG in AD in clinical trials.

First Author	ADAS-Cog	MMSE	CDR-SB	Other	Aβ_40_ in Plasma	Aβ_42_ in Plasma	Aβ_40_ in CSF	Aβ_42_ in CSF	Test Item	Result
Kile [59,61]	↓ LMIC, 1 year, more favourable	↑ LMIC	N	O	O	O	O	O	APCV	↓ L-MCI were most pronounced
Kile [55]	N	N	N	N	N	N	N	N	SUVR; amyloid retinal deposits	↓ (3/5); ↓ (all)
Kasai [60]	↓ 3/5)	↑ (3/5)	O	FAST (O)	↑ (3/5, in jugular-plasma)	↑ (3/5, in jugular-plasma)	N	O	Aβ deposition measured by PIB-PET DVR map	N
Relkin [57]	O	N	N	ADCS-ADL, ADCS-CGIC, NPI (O)	N	↓	N	N	APCV	O
Dodel [44]	N	N	N	N	N	N	N	N	Brain volume atrophy rate	O
Relkin [58]	O	↑ after 9 months of renewed IVIG infusions	O	O	↑	↑	↓	↓	N	N
Dodel [56]	↓ a slight improvement	↑ a slight improvement	N	N	↑		↓		N	N
Dodel [37]	N	N	N	N	↑		↓		N	N

Note: ↑/↓ = statistically significant increase/decrease (*p* < 0.05). O = no significant difference. N = not reported. ADAS-Cog: Alzheimer’s Disease Assessment Scale-Cognitive Subscale. ADCS-ADL: Alzheimer’s Disease Cooperative Study-Activities of Daily Living. ADCS-CGIC: Alzheimer’s Disease Cooperative Study-Clinical Global Impression of Change. CDR-SB: Clinical Dementia Rating Scale Sum of Boxes. APCV: annualized percent change in ventricular volume. MMSE: Mini-Mental State Examination. FAST: Functional Assessment Staging. NPI: Neuropsychiatric Inventory. SUVR: standard uptake value ratio. PIB: Pittsburgh Compound-B. PET: Positron Emission Tomography. DVR: Distribution Volume Ratio.

**Table 5:** The efficacy of IVIG and PE treatment in AD in clinical trials.

Group	ADCS-ADL	ADAS-Cog	CDR-sb	ADCS-CGIC	Aβ_40_ (CSF)	Aβ_42_ (CSF)	T-Tau (CSF)	P-Tau (CSF)	RAVLT 1	RAVLT 3	RAVLT 2, 4, 5	PVF	SVF	SDMT	QoL-AD	72 h AE
PE + low-albumin	O	O	Significantly less ↓	S	N	N	N	N	↓	S	O	S	S consistent improvement	S	N	16.40%
PE + low-albumin + IVIG			Significantly less ↓	S	N	N	N	N	Less ↓ at month 2 *p* = 0.02	↑ at month 2 *p* = 0.04		↑ at month 14 *p* = 0.02	Less ↓ *p* > 0.05	Increased	N	15.70%
PE + high-albumin + IVIG			Significantly less ↓	S	N	N	N	N	Less ↓ at month 2, 12, 14 *p* = 0.03, 0.08, 0.004	↑ at month 2, 14 *p* = 0.01, 0.01		↑ consistent improvement	Less ↓ *p* > 0.05	Increased at month 14 *p* = 0.03	N	18.80%
s	52% less ↓ *p* = 0.03 at 14 month	66% less ↓ *p* = 0.06 at 14 month	65–71% less ↓ *p* = 0.02~0.1	S *p* < 0.01~0.02	S	S	S	S	Less ↓ at month 2, 6, 14 *p* = 0.06, 0.08, 0.07	↑ at month 2 *p* = 0.04		↑ at month 9, 12, 14 *p* = 0.05, 0.09, 0.007	Less ↓ at month 14 *p* = 0.03	↑ at month 14 *p* = 0.05	↑ at month 14 *p* = 0.02	N
	Placebo group	↓	↓	↓	↓	S	↓	↑ in moderate AD, ↓ in mild AD	↓	S		↓	↓	↓	S	4.10%

Note: ↑/↓ = increase/decrease. O = no significant difference. N = not reported. S = stabilization.

**Table 6:** The safety of IVIG in AD in clinical trials.

Author	Publication Year	Experimental Subjects	Safety
Kile [59,61]	2021, 2015	50 MCI due to AD patients	no drug-related adverse events
Kasai [60]	2017	5 patients with AD	no drug-related adverse events
Relkin [57]	2017	390 patients with mild to moderate AD	frequency of nonserious adverse events was decreased
Gelmont [68]	2016	383 patients with mild to moderate AD	no unexpected safety findings
Arai [69]	2014	16 patients with mild to moderate AD	IVIG was safe and well tolerated
Dodel [44]	2013	89 patients with mild to moderate AD	IVIG may have an acceptable safety profile
Relkin [58]	2009	8 patients with mild AD	well tolerated
Dodel [56]	2004	5 patients with AD	well tolerated

**Table 7:** GRADE Summary of Findings for IVIG intervention in patients with AD or MCI. Patient or population: patients with AD or MCI; intervention: IVIG; comparison: placebo or baseline (pre-treatment).

Outcomes	No. of Studies (Design)	Total Participants (N)	Certainty of Evidence (GRADE)	Key Findings
**Cognitive Function** (ADAS-Cog, MMSE)	9 studies (RCTs and Non-randomized)	1055	⊕⊕◯◯ **Low** ^a^	Majority of studies showed a trend toward stabilization, but the largest Phase III trial (*n* = 390) failed to meet primary endpoints.
Plasma Biomarkers (Aβ40, Aβ42)	6 studies	509	⊕⊕⊕◯ **Moderate** ^b^	Significant increase in plasma Aβ levels post-infusion, supporting the “peripheral sink” hypothesis.
CSF Biomarkers (Aβ, Tau)	4 studies	111	⊕⊕◯◯ **Low** ^c^	Decrease in CSF Aβ observed in small cohorts, but results varied across different IVIG products.
Brain Atrophy (APCV, Ventricular Volume)	3 studies	529	⊕⊕◯◯ **Low** ^d^	Potential reduction in brain atrophy rate (ventricular enlargement) observed in MCI and mild AD subgroups.
Safety and ARIA (Adverse Events)	9 studies	1454	⊕⊕⊕⊕ **High** ^e^	IVIG demonstrated a superior safety profile with no significant risk of ARIA compared to MABs.

Note: a = Downgraded due to inconsistency and risk of bias: several included studies were single-arm, open-label trials. Furthermore, the results of the large-scale Phase III trial [57] were inconsistent with findings from earlier, smaller-scale pilot studies. b = Downgraded due to imprecision: although the direction of effect was generally consistent across studies, the concentrations of Aβ-related antibodies varied significantly between IVIG products from different manufacturers and batches. c = Downgraded due to imprecision: a limited number of studies reported cerebrospinal fluid (CSF) biomarkers, resulting in an insufficient aggregate sample size for a high-certainty conclusion. d = Downgraded due to risk of bias: high rates of missing data and participant attrition were observed in certain neuroimaging studies (e.g., Kile [59] was assessed as having a high risk of bias due to incomplete outcome data). e = No downgrading: all clinical trials consistently demonstrated a favorable safety profile for IVIG across diverse patient cohorts, providing robust and reliable evidence.

## Data Availability

The dataset supporting the conclusions of this article is included within the article.

## Supplementary Materials

The following supporting information can be downloaded at: https://www.mdpi.com/article/10.3390/ijms27052275/s1.

## Abbreviations

AD	Alzheimer’s disease
ADAS-Cog	Alzheimer’s Disease Assessment Scale-Cognitive Subscale
ADCS-ADL	Alzheimer’s Disease Cooperative Study-Activities of Daily Living
ADCS-CGIC	Alzheimer’s Disease Cooperative Study-Clinical Global Impression of Change
AE	Adverse event
APCV	Annualized percent change in ventricular volume
ARIA	Amyloid-Related Imaging Abnormalities
ARIA-E	Amyloid-Related Imaging Abnormalities-edema
ARIA-H	Amyloid-Related Imaging Abnormalities-hemorrhage
Aβ	amyloid-beta
CDR-SB	Clinical Dementia Rating Scale Sum of Boxes
CSF	cerebrospinal fluid
CT	Computed Tomography
DVR	Distribution Volume Ratio
FAST	Functional Assessment Staging
IVIG	intravenous immunoglobulin
MABs	monoclonal antibodies
MMSE	Mini-Mental State Examination
MRI	Magnetic Resonance Imaging
NPI	Neuropsychiatric Inventory
PE	Plasma exchange
PET	Positron Emission Tomography
PIB	Pittsburgh Compound-B
PVF	Phonetic Verbal Fluency
QoL-AD	quality of life-Alzheimer’s Disease
RAGE	Receptor for Advanced Glycation Endproducts
RAVLT	Rey Auditory Verbal Learning Test
SDMT	Symbol Digit Modalities Test
sLRP1	soluble low-density lipoprotein receptor-related protein 1
SUVR	standard uptake value ratio
SVF	Semantic Verbal Fluency
